# Distribution of Therapeutic Efficacy of Ranunculales Plants Used by Ethnic Minorities on the Phylogenetic Tree of Chinese Species

**DOI:** 10.1155/2022/9027727

**Published:** 2022-01-12

**Authors:** Da-Cheng Hao, Yulu Zhang, Chun-Nian He, Pei-Gen Xiao

**Affiliations:** ^1^Biotechnology Institute, School of Environment and Chemical Engineering, Dalian Jiaotong University, Dalian 116028, China; ^2^Institute of Molecular Plant Science, University of Edinburgh, Edinburgh EH9 3BF, UK; ^3^Institute of Medicinal Plant Development, Chinese Academy of Medical Sciences, Beijing 100193, China

## Abstract

The medicinal properties of plants can be evolutionarily predicted by phylogeny-based methods, which, however, have not been used to explore the regularity of therapeutic effects of Chinese plants utilized by ethnic minorities. This study aims at exploring the distribution law of therapeutic efficacy of Ranunculales plants on the phylogenetic tree of Chinese species. We collected therapeutic efficacy data of 551 ethnomedicinal species belonging to five species-rich families of Ranunculales; these therapeutic data were divided into 15 categories according to the impacted tissues and organs. The phylogenetic tree of angiosperm species was used to analyze the phylogenetic signals of ethnomedicinal plants by calculating the net relatedness index (NRI) and nearest taxon index (NTI) in R language. The NRI results revealed a clustered structure for eight medicinal categories (poisoning/intoxication, circulatory, gastrointestinal, eyesight, oral, pediatric, skin, and urinary disorders) and overdispersion for the remaining seven (neurological, general, hepatobiliary, musculoskeletal, otolaryngologic, reproductive, and respiratory disorders), while the NTI metric identified the clustered structure for all. Statistically, NRI and NTI values were significant in 5 and 11 categories, respectively. It was found that *Mahonia eurybracteata* has therapeutic effects on all categories. iTOL was used to visualize the distribution of treatment efficacy on species phylogenetic trees. By figuring out the distribution of therapeutic effects of Ranunculales medicinal plants, the importance of phylogenetic methods in finding potential medicinal resources is highlighted; NRI, NTI, and similar indices can be calculated to help find taxonomic groups with medicinal efficacy based on the phylogenetic tree of flora in different geographic regions.

## 1. Introduction

The vascular plants, thriving in diverse environments, include mosses, liverworts, ferns, gymnosperms (conifers), and angiosperms (flowering plants) [1]. Among these taxonomic groups, angiosperms are the latest in evolution, but have the most species; in accordance with their strong environmental adaptability, a majority of phytometabolites with known/potential services to humankind is from angiosperms [2]. There are more than 10,000 extant genera and more than 200,000 species of angiosperms in our planet, accounting for more than half of all plant species. There are more than 2,700 genera and more than 30,000 known species of angiosperms in China, which represents one of the most species-rich areas of the world. The survival and health of human beings are closely related to angiosperms; more than 50 ethnic minorities in China have accumulated rich experiences in collecting and applying botanical drugs [3], especially those from angiosperms. In China, there are 10,027 kinds of medicinal angiosperms (including the infra-species taxonomic units) [4], accounting for 90% of Chinese medicinal species. Most of the traditional Chinese medicine (TCM) and ethnomedicine/folk medicine are from angiosperms, but the quantitative study exploring the association between Chinese angiosperm phylogeny and various therapeutic effects of phytomedicine is rare [5, 6], and the traditional medicinal knowledge of China ethnic minorities has not been elaborated within the context of plant phylogeny. The continued globalization of herbal medicine, badly controlled exploitative practices, and inadequate conservation efforts are pushing many precious medicinal plants to the edge of extinction. Meanwhile, traditional therapeutic knowledge is being eroded due to changing lifestyles, awareness, social transformations, and acculturation [7]. Phylogenetic methods constantly reveal the predictive ability of traditional medical knowledge in bioprospecting and pharmaceutical resource discovery [8–11], which, however, has not been systematically utilized in scrutinizing and expanding Chinese ethnomedicinal resources. We hypothesize that the traditionally used ethnomedicinal plants in China can be quantitatively and phylogenetically explored, which afford generally applicable clues for bio-screening medicinal flora and discovering alternative/complementary medicine.

The medicinal value of plants is an essential part of ecosystem services. The botanical chemo diversity, based on plant biodiversity [6], is the cornerstone of pharmacotherapy diversity of plants and plant-based products. In many areas, medicinal herbs were the main approaches of traditional therapies, which were considered the main lifeline and recurrently were the first and/or only choice [7]. About 80% of plant species used globally as drug sources have an ethnomedical use identical or related to the current use of active elements of the plant [12]. Therefore, ecosystem services are closely related to human well-being. The rapid growth of population and public health crisis faced by many regions, especially in developing countries, make it imperative to find alternative and complementary phytomedicine resources [13]. Due to the lack of awareness of environmental protection, human activities have caused severe damage to the ecological environment, in which numerous factors play an important role in the formation and accumulation of effective components of medicinal plants [14]. In view of the importance of ethnomedicinal plants in combating diseases and improving primary healthcare, it is a must to strengthen the protection of medicinal plant resources, and the most fundamental is to protect the ecological environment.

Phylogenetic approaches may help in finding resources for natural and eco-friendly therapy [15]. China is an ideal country to analyze the phylogenetic model of medicinal plants, because of the diversity and good regional phylogeny of Chinese traditional medicinal plants [16–18]. Many secondary metabolites of ethnomedicinal plants of Ranunculales, for example, alkaloids, terpenoids, and flavonoids, have unique functions and biological activities, providing a probability premise for appropriately targeting the dysfunctional organs of the human body [19, 20]. For example, berberine extracted from *Coptis* of Ranunculaceae had a synergistic anticancer effect by inducing apoptosis and inhibiting cell proliferation of esophageal cancer cells [21]. Five benzylisoquinoline alkaloids (columbamine, palmatine, dauricine, jatrorrhizine, and berberine; BIAs) from *Dichocarpum* of Ranunculaceae had the *in vitro* inhibitory activity against acetylcholinesterase [22, 23]. Therefore, taking the order Ranunculales as a representative, we aim at studying the distribution pattern of Chinese ethnomedicinal plants on the phylogenetic tree by combining the species phylogenetic tree of Chinese angiosperms and therapeutic data of ethnomedicinal plants at the species level.

## 2. Materials and Methods

### 2.1. Efficacy Arrangement of Ethnomedicinal Plants

In the Chinese Dictionary of Ethnic Medicine [3], 180 ethnomedicine books and 1,118 journal papers published by various ethnic groups in the past 40 years were cited. It is confirmed that there are 7,736 kinds of drugs used by 53 ethnic minorities in China, including 7,022 phytomedicines, 551 animal drugs, and 163 mineral drugs, among which 656 are also recorded in Chinese Pharmacopoeia. The data of skin efficacy were also searched in NCBI PubMed and China National Knowledge Infrastructure (https://cnki.net/). The therapeutic effects of 551 medicinal species, including 104 varieties, in five species-rich families were collected and summarized (Table S1), which involve 300 Ranunculaceae species (54.4% of all species), 69 Berberidaceae species (12.5%), 42 Menispermaceae species (7.6%), 14 Lardizabalaceae species (2.5%), and 126 Papaveraceae species (22.9%). All curative effects were divided into 15 categories: poisoning/intoxication, circulatory diseases, gastrointestinal diseases, nervous system diseases, eye diseases, other/general diseases, hepatobiliary diseases, musculoskeletal diseases, oral diseases, ear/nose/throat diseases, pediatric diseases, reproductive system diseases, respiratory diseases, skin diseases, and urinary diseases. The therapeutic efficacy of ethnomedicinal plants was coded with binary characters: when a species has the efficacy of treating specific diseases, it is 1; otherwise, it is 0.

### 2.2. Phylogenetic Tree of Ranunculales

The phylogenetic tree used in this study is full_tree_461.tre (http://www.darwintree.cn/resource/Nature2018/), which is the overall phylogenetic diversity model of China angiosperms constructed by Lu et al. [17] at the species level. The phylogenetic data of 92% angiosperms in Chinese flora are utilized in this large tree, which is a nearly complete species-level phylogenetic tree including 26,978 species. The Ranunculales subtree was extracted from the above large tree, including Ranunculaceae, Berberidaceae, Menispermaceae, Lardizabalaceae, Circaeasteraceae, Papaveraceae, and Eupteleaceae; the scientific names of species are mainly based on the Flora of China (http://www.iplant.cn/frps). The R packages Picante [24] and Ape (https://cran.r-project.org/web/packages/ape/) were used to generate the subtree with the following commands: sample <- read.csv(“species.csv,” header = T), tree <- read.tree(“full_tree_461.tre”), tip.all <-tree$tip.label, tip.not<- tip.all[!tip.all%in%(sample[,1])], length(tip.not), tr.new <- drop.tip(tree,tip.not), write. tree (tr.new, “phylo_zhang_MGM.tree”), read.tree(“phylo_zhang_MGM.tree”). iTOL v6 (https://itol.embl.de/) was used to draw and visualize the phylogenetic tree of Ranunculales.

### 2.3. Statistics and Calculation of Phylogenetic Distribution

The NRI (net relatedness index) was used to quantify the mean pairwise phylogenetic distance (MPD) of ethnomedicinal plants [10], which designates the dispersion of medicinal uses toward the root of phylogeny. The NTI (nearest taxon index) is a standardized index to measure the average phylogenetic distance between each sample and its nearest taxon, which can be used to calculate the mean nearest taxon distance (MNTD); NTI describes the dispersion of medicinal uses toward the tips of phylogeny [9]. The positive values of these two indicators suggest the phylogenetic aggregation of medicinal species, whereas negative values indicate that species with the same therapeutic use are dispersed in the phylogenetic tree [9]. The observed patterns of species distribution were compared with the expected patterns to quantitatively assess whether the values of NRI and NTI were statistically significant (*p* < 0.05). The calculation formula is as follows:

(1) $$\begin{array}{c} \text{NRI}=-1\times \frac{{\text{MPD}}_{\text{obs}}-{\text{MPD}}_{\text{null}}}{\text{sd}\left({\text{MPD}}_{\text{null}}\right)}, \end{array}$$

where MPD_obs_ is the observed MPD, MPD_null_ is the expected MPD of the randomized group, and sd(MPD_null_) is the standard deviation of MPD_null_. The NTI is given as

(2) $$\begin{array}{c} \text{NTI}=-1\times \frac{{\text{MNTD}}_{\text{obs}}-{\text{MNTD}}_{\text{null}}}{\text{sd}\left({\text{MNTD}}_{\text{null}}\right)}, \end{array}$$

where MNTD_obs_ denotes the observed MNTD, whereas MNTD_null_ denotes the expected MNTD of the randomized group, and sd(MNTD_null_) denotes the standard deviation of MNTD_null_ [25].

The functions ses. mpd and ses. mntd of Picante package [24] were used to calculate the NRI and NTI, respectively. The “taxa. labels” was used as the null model, which shuffles the distance matrix labels across all taxa included in the distance matrix with 999 runs; “mpd.obs.p” is the *p* value of observed MPD vs. null communities (= mpd.obs.rank/(runs + 1)). R language codes for the construction of tree as well as calculation of the NRI and NTI are shown in the Supplementary text and are available from the authors upon request.

## 3. Results

### 3.1. Distribution of Therapeutic Efficacy in the Order Ranunculales

**Figure 1:**
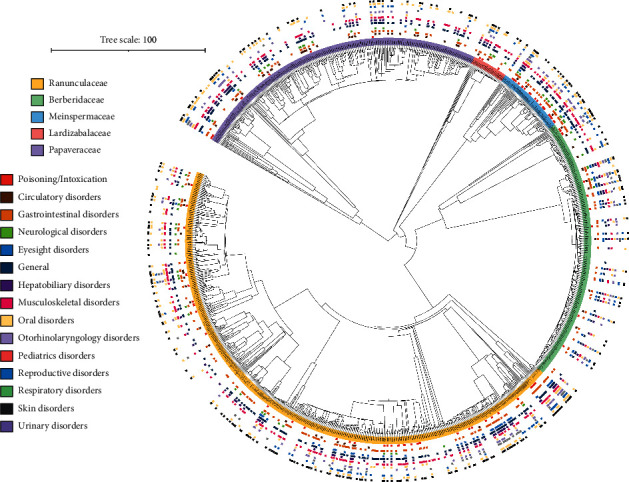
Distribution of 15 therapeutic efficacies of Chinese ethnomedicinal species on the phylogenetic tree of Chinese angiosperms. There are 1,519 Chinese Ranunculales species in this large tree, including 725 Ranunculaceae species (yellow in the inner circle), 306 Berberidaceae species (green), 78 Menispermaceae species (blue), 44 Lardizabalaceae species (red), 364 Papaveraceae species (purple), one Eupteleaceae species, and one Circaeasteraceae species. From the inside to the outside of the outer circle are poisoning, circulatory diseases, gastrointestinal diseases, nervous system diseases, eye diseases, other/general diseases, hepatobiliary diseases, musculoskeletal diseases, oral diseases, ear/nose/throat diseases, pediatric diseases, reproductive system diseases, respiratory diseases, skin diseases, and urinary diseases.

In Chinese ethnomedicinal plants of Ranunculales (Figure 1), the highest number of species (435, 78.9% of all medicinal species) is used for the treatment of general/other diseases, followed by musculoskeletal disease (389), skin disease (348), and gastrointestinal disease (343). The least species are used to treat pediatric diseases (73, 13.2% of species). The NRI designates the dispersion of medicinal uses toward the root of phylogeny [9]. A clustered structure was suggested by NRI values (>0) for eight categories, that is, poisoning/intoxication (e.g., *Anemone*, Ranunculaceae; Tables S1 and S2), circulatory disease (*Corydalis*, Papaveraceae), gastrointestinal disease (*Aconitum*, Ranunculaceae), eye disease (*Thalictrum*, Ranunculaceae), oral disease (*Aconitum*), pediatric disease (*Thalictrum*), skin disease (*Aconitum*), and urinary disease (*Clematis*, Ranunculaceae). The other seven categories were of overdispersion (NRI < 0), although it still can be seen that there are more therapeutic species against nervous system diseases, other/general diseases, hepatobiliary diseases, musculoskeletal diseases, and respiratory diseases in *Corydalis* (Table S1), and species against ear/nose/throat diseases and reproductive system diseases are concentrated in *Berberis* (Berberidaceae) and *Clematis*, respectively. The NTI describes the dispersion of medicinal uses toward the tips of phylogeny [9], and the clustered structure was suggested for all 15 categories (NTI > 0). In a statistical test, the NRI suggested that five categories, that is, eye diseases, oral diseases, ear/nose/throat diseases, skin diseases, and urinary diseases, are of cluster with statistical significance (*p* < 0.05; Table 1), whereas the NTI suggested 11 categories with statistically significant cluster (*p* < 0.05). Both the NRI and the NTI suggest eye diseases, oral diseases, skin diseases, and urinary diseases as statistically significant clustered.

It is found that 66 Ranunculales species, accounting for 12% of species, have therapeutic effects on no less than ten categories of diseases (Table 2). For example, *Mahonia eurybracteata* of Berberidaceae showed medicinal utilities against all 15 categories. In Dong medicine, the whole plant is used against rheumatic pain, bruise and swelling pain, stomach cold pain, snake/centipede bite, and the like [3]; in Tu Jia medicine, its efficacy is the same as that of *M. bealei*. Three multipurpose species covering 14 therapeutic categories are M. *bealei*, *M. fortunei*, and *M. gracilipes*; no wonder the Chinese name of the genus *Mahonia* literally means “the top ten credits.” Three *Tinospora* species, two *Berberis* species, and two *Aconitum* species have 13 therapeutic categories(Table 2).

### 3.2. Distribution of Therapeutic Efficacy in Five Major Families of Ranunculales

Among Chinese ethnomedicinal plants of Ranunculaceae (buttercup family), the highest number of species (233, 77.7% of species; Table S2) is used for the treatment of general/other diseases, followed by musculoskeletal diseases (199), skin diseases (199), and gastrointestinal diseases (188). The least species are used to treat circulatory diseases (27, 9% of species). A clustered structure was suggested by NRI values (>0) for 10 categories, whereas the other five categories were of overdispersion (NRI < 0). The clustered structure was suggested by NTI > 0 for 13 categories. In the statistical test, the NRI suggested that six categories, that is, circulatory disease, nervous system diseases, eye diseases, pediatric diseases, reproductive system diseases, and urinary diseases, are of cluster with statistical significance (*p* < 0.05; Table S2), whereas the NTI suggested six categories with statistically significant cluster (*p* < 0.05). Both the NRI and the NTI suggest eye diseases, reproductive system diseases, and urinary diseases as significantly clustered.

In Chinese ethnomedicinal plants of Berberidaceae (barberry family), the highest number of species (56, 81.1% of species; Table S2) is used for the treatment of general/other diseases, followed by gastrointestinal diseases (55), musculoskeletal diseases (51), and skin diseases (48). The least species are used to treat nervous system diseases (9, 13.0% of species). A clustered structure was suggested by NRI values (>0) for seven categories, while the other eight categories were of overdispersion (NRI < 0). The clustered structure was suggested by NTI > 0 for 12 categories. In the statistical test, the NRI suggested that four categories, that is,, gastrointestinal diseases, eye disease, hepatobiliary diseases, and ear/nose/throat diseases, are of cluster with statistical significance (*p* < 0.05; Table S2), whereas the NTI suggested no category with statistically significant cluster (*p* > 0.05).

Among Chinese ethnomedicinal plants of Menispermaceae, the highest number of species (34, 80.9% of species; Table S2) is used for the treatment of gastrointestinal/musculoskeletal diseases, followed by general/other diseases (33) and skin diseases (27). Only two species are used to treat pediatric diseases. A clustered structure was suggested by NRI values (>0) for 13 categories, whereas the other two categories were of overdispersion (NRI < 0). The clustered structure was suggested by NTI > 0 for 13 categories. In the statistical test, the NRI suggested that two categories, that is,, other/general diseases and pediatric diseases, are of cluster with statistical significance (*p* < 0.05; Table S2), whereas the NTI suggested seven categories with statistically significant cluster (*p* < 0.05). Both the NRI and the NTI suggest other/general diseases and pediatric diseases as significantly clustered.

In Chinese ethnomedicinal plants of Lardizabalaceae, the highest number of species (14, 100% species; Table S2) is used for the treatment of musculoskeletal diseases, followed by other/general diseases (12) and skin/respiratory/urinary diseases (10 of each). No species is used to treat eye/oral diseases. A clustered structure was suggested by NRI values (>0) for four categories, that is, poisoning/intoxication, nervous system disease, ear/nose/throat disease, and urinary disease, whereas the other seven categories were of overdispersion (NRI < 0). The clustered structure was suggested by NTI > 0 for four categories. In the statistical test, the NRI suggested that nervous system disease is of cluster with statistical significance (*p* < 0.05), whereas the NTI suggested no statistically significant category.

Among Chinese ethnomedicinal plants of Papaveraceae (poppy family), the highest number of species (101, 80.1% of species) is used for the treatment of general/other diseases, followed by musculoskeletal diseases (91), respiratory diseases (83), and skin diseases (64). The least species are used to treat eye/pediatric diseases (eight of each). A clustered structure was suggested by NRI values (>0) for two categories, that is, circulatory and nervous system diseases, whereas the other 13 categories were of overdispersion (NRI < 0; Table S2). The clustered structure was suggested by NTI > 0 for 14 categories. In the statistical test, the NRI suggested that circulatory and nervous system diseases are of cluster with statistical significance (*p* < 0.05), whereas the NTI suggested circulatory and hepatobiliary diseases with statistically significant cluster (*p* < 0.05). Both the NRI and the NTI suggest circulatory disease as significantly clustered.

## 4. Discussion

By utilizing NRI and NTI in R language, this study presents the first quantitative evidence of the association between angiosperm phylogeny and therapeutic efficacy of Chinese ethnomedicinal species. These indices were calculated for 14 categories of diseases to examine the phylogenetic clustering of TCM plants in China [5]. However, this study focused on legal medicinal plants of China, and it is unknown how many medicinal plants used by ethnic minorities were included. China is a multi-ethnic country, in which the Han population is the largest, accounting for about 92% of the total population. The total population of the other 55 ethnic groups is ∼115,145,902, accounting for about 8% of the total population, so they are called ethnic minorities. At least 7,022 botanical species are used medicinally by China ethnic minorities [3], representing an enormous space for bioprospecting and drug development. Here, the order Ranunculales is chosen for the case study of traditional medicinal knowledge of China ethnic minorities, which belongs to the basal eudicots and is evolutionarily older than other eudicots [26, 27]. Basal eudicots radiate for maximum disorders [5]; accordingly, various Ranunculales species have been employed in TCM, traditional oriental medicine, and Western folk medicine for diverse ailments [19, 28]. Widely known Ranunculales members, for example, blue cohosh, black seed, poppies, barberries, and buttercups, are being studied for their pharmacological activities via integrative omics techniques and from the systems perspective. Ranunculales contributes plenty of botanical extracts with therapeutic efficacy and/or health-promoting effects. As there are more than 4,000 and more than 1,500 Ranunculales species in the world and China, respectively, and novel Ranunculales taxa, that is, *Dichocarpum lobatipetalum* and *D. malipoense* [29], are being discovered, there are still vast biological and chemical space to be prospected in this well-known medicinal order.

### 4.1. Ranunculaceae

At the order level, the NRI and NTI revealed eight and 15 therapeutic categories with clustered structure, respectively, but scrutinizing each family could help gain deeper understanding of the therapeutic values of genera/species and narrow down bio-screening. Ranunculaceae could be the most important medicinal family in Ranunculales and even in basal eudicots; in TCM, up to 63% of Ranunculaceae medicinal species have utilities in musculoskeletal disorders [5], followed by hepatic disorders (56%) and circulatory disorders (42.2%). In the present study, 199 out of 300 Ranunculaceae medicinal species are used by China ethnic minorities against musculoskeletal diseases and skin diseases (Table S2), followed by 188 for gastrointestinal disease and 84 for hepatobiliary disease. Berberidaceae is the second important medicinal family in Ranunculales. In TCM, 81.5% of Berberidaceae medicinal species are used against eyesight disorders [5], followed by hepatic disorders (80.8%) and digestive disorders (78.6%). In contrast, 55 and 51 out of 69 Berberidaceae medicinal species are used by China ethnic minorities against gastrointestinal and musculoskeletal diseases, respectively (Table S2), followed by 48 for skin disease and 42 for eye disease. These results illustrate the significant difference of disease spectrum and medication experience between TCM and traditional medicine of ethnic minorities; the latter is a precious intangible cultural heritage. In order to avoid rapid loss, its protection, sorting, and inheritance should be vigorously strengthened.

Ranunculaceae, mostly herbs and some of which are small shrubs or woody vines, includes about 60 genera and 2,200 species [30]. Plants of this family are distributed worldwide, mainly in the temperate region of the northern hemisphere. Forty-two genera and around 720 species are distributed throughout China, most of which are in the southwest mountainous region. In this study, up to nine therapeutic categories showed a clustered structure in Ranunculaceae (Tables S1 and S2, Figure 1). Some species of genera *Clematis*, *Coptis*, and *Ranunculus* are commonly used by some ethnic minority groups in China to combat against ocular inflammation, infection, keratopathy, and cataract, among others [3]. *Cimicifuga*, *Clematis,* and *Coptis* are often used to treat reproductive system diseases, especially gynecological and obstetric ones. *Clematis*, *Nigella,* and *Thalictrum* are salient in the treatment of urinary inflammation, infection, and calculi. The therapeutic species against skin/gastrointestinal diseases are relatively concentrated in *Aconitum*, *Clematis,* and *Delphinium*; the circulatory disease targeting species are concentrated in *Aconitum* and *Clematis*. Thirty-four Ranunculaceae species are used ethnomedicinally against nervous system disease (Tables S1 and S2). We also noted the folk medicine use of *Dichocarpum auriculatum* (Ranunculaceae) in Sichuan Province against sow mania (epilepsy of sow) [23], which is not recorded in the Dictionary of Chinese Ethnic Medicine [3], as this book mainly reflects the traditional medicine knowledge of Chinese ethnic minorities. Accordingly, five BIAs of *D. auriculatum* had the *in vitro* inhibitory activity against acetylcholinesterase [22].

Chemical components of Ranunculaceae include several representative groups: BIA, ranunculin, triterpenoid saponin, and diterpene alkaloid,. [30]. Ranunculin and magnoflorine coexist in some genera. Our early ethnopharmacological investigation showed that the most frequent ethnopharmacological uses, mainly in Han Nationality, are heat-clearing and detoxification, ulcer disease and sore, anti-microbe, and anti-inflammation [31]; the most studied bioactivities are anticancer/cytotoxic, antimicrobial, and anti-inflammatory activities. These results cross-validate the utility of traditional medicinal knowledge in bioprospecting and contemporary phytotherapy research.

### 4.2. Berberidaceae

In other four species-rich families of Ranunculales, hot nodes/clades for drug discovery and development are also identified by NRI/NTI (Tables S1 and S2, Figure 1). In Berberidaceae (17 genera, 650 species), four therapeutic categories showed a clustered structure (Tables S1 and S2, Figure 1). Some species of genera *Berberis*, *Dysosma,* and *Mahonia* are especially useful in the treatment of gastrointestinal, eye, and ear/nose/throat diseases [3]. The therapeutic species against eye and hepatobiliary diseases are relatively concentrated in *Berberis* and *Mahonia*, which are close to each other phylogenetically. Twenty-six *Berberis* species and 11 *Mahonia* species are used by China ethnic minorities against various eye diseases. For example, in Mongolian medicine, *B. amurensis* and *B. vernae* are used for sore red swollen eyes, acute conjunctivitis, and ocular leukoplakia; in Tibetan medicine, the flower juice of *B. vernae* is used to drop eyes to treat eye diseases. *Mahonia bealei* is used by Bu Yi, Mao Nan, Miao, Mu Lao, and Yi Nationality against acute conjunctivitis. In Ge Lao medicine, the water decoction of *M. bealei* root is used to wash eyes for treating ocular itching and lacrimation. It happens that *Berberis* is similarly used in other traditional medicine systems. For example, in far-west Nepal, *B. asiatica* is frequently used for eye problems [32]; in Islamic traditional medicine, different parts of *B. vulgaris* and *B. integrrima* are prescribed for skin, liver, stomach, kidney, and eye problems [33]. In the Indian and European systems of traditional medicine, *Berberis* is used for curing eye disease, fever, jaundice, rheumatism, vomiting during pregnancy, kidney and gall balder stone, and such like [34]. Interestingly, the utility of *Mahonia* in eye disease is not salient in these traditional medicine systems except in TCM and traditional medicine of China ethnic minorities. There are about 100 species in the genus *Mahonia*, which are distributed in Central/North America and Asia. The unique ethnopharmacological uses in China ethnic minorities might contribute much in the search for new sources of pharmaceuticals.

*Berberis* and *Mahonia* contain mainly BIAs, for example, berberine, palmatine, jatrorrhizine, columbamine (Protoberberine type), magnoflorine (aporphine type), particularly a higher content of bisbenzylisoquinoline (BBI) alkaloids berbamine [35], isotetrandrine, and oxyacanthine. Phenolics, flavonoids, and tannins are abundant in *Berberis* [36]. These compounds constitute the material basis of traditional/modern therapeutic efficacy. Berberine is the principal component of many medicinal plants, for example, *Coptis chinensis*, *Hydrastis canadensis* [37], *Berberis vulgaris* [38], *B. aristate* [39], and *Mahonia bealei*, which could partially explain the shared therapeutic efficacy between Ranunculaceae and Berberidaceae. It should be noted that although the genus *Epimedium* was not suggested by the NRI and NTI, eight *Epimedium* species are ethnomedicinally used for various diseases [3], especially reproductive disorders, musculoskeletal disorders, and pediatrics disorders. This illustrates the complementarity, rather than substitutability, of the phylogenetic approach in bioprospecting.

### 4.3. Menispermaceae, Lardizabalaceae, and Papaveraceae

In Menispermaceae (65 genera, >350 species), seven categories showed a clustered structure. Two *Tinospora* species are ethnomedicinally used for pediatric diseases, whereas *Cocculus*, *Cyclea,* and *Stephania* species are traditionally used for various general/difficult-to-classify diseases. BBI alkaloids are abundant in Berberidaceae and Menispermaceae [28]. BBI alkaloids, morphine alkaloids, aporphine alkaloids, syringaresinol, and aristolochic acid І could be marker compounds of Menispermaceae and could be responsible for the efficacy of *Stephania* and *Tinospora* against poisoning, ear/nose/throat diseases, and skin diseases, among others (Tables S1 and S2). In TCM, the root of *Stephania tetrandra* is used to treat arthralgia caused by rheumatism, wet beriberi, dysuria, eczema, and inflamed sores [40], which is similar to the usage of ethnic minorities. In Southeast Asia, the stems, leaves, and tubers of *Stephania rotunda* are used in the Cambodian, Lao, Indian, and Vietnamese folk medicine systems for years to treat a wide range of ailments [41], including asthma, headache, fever, and diarrhea. *Tinospora cordifolia* is used in folk and Ayurvedic medicines throughout India [42]. This plant contains many pharmaceutical compounds such as alkaloids, diterpenoid lactones, glycosides, steroids, sesquiterpenoid, and phenolics, which make it antidiabetic, antipyretic, anti-inflammatory, antioxidant, hepatoprotective, and immuno-modulatory. The phytochemistry and pharmacology of *T. cordifolia* are analogous to that of *Tinospora* species used by China ethnic minorities [3].

The small family Lardizabalaceae has nine genera and 50 species; only species against nervous system disease showed a cluster, that is, the genus *Stauntonia* [3], *Stauntonia brachyanthera*, *S. chinensis*, *S. obovatifoliola*, and *S. yaoshanensis* are ethnomedicinally used to alleviate trigeminal neuralgia. Recent pharmacological investigations revealed the anti-inflammatory activity of *S. hexaphylla* [43] and *S. brachyanthera* [44], but not nervous system effects; thus, our results could be valuable for future research direction. Ten out of 14 Lardizabalaceae species are used by China ethnic minorities against urinary diseases (Table S2). For example, in Dong medicine, the rattan stems of *Akebia quinata* are used against edema, adverse urination, blennorrhagia, and urinary stone [3]; in Mongolian medicine and Miao medicine, the fruit of *A. quinata* is used for adverse urination; in Tu Jia medicine, the fruit of *A. quinata* is used for rough voidings of reddish urine, and its pericarp and seeds benefit the kidney and reduce swelling. *A. trifoliata* and *A. trifoliata var. australis* are similarly used by ethnic minorities. It is found that akebia saponin D ameliorated kidney injury and exerted anti-inflammatory and anti-apoptotic effects in diabetic nephropathy by activating NRF2/HO-1 and inhibiting NF-*κ*B pathway [45]. More pharmacological explorations of Lardizabalaceae components are warranted, so as to validate the ethnopharmacological uses and expand new application space.

The BIA biosynthetic pathway underwent the parallel evolution between two basal eudicot orders Ranunculales and Proteales [46], which diverged 122 million years ago (MYA). Berberine, belonging to the protoberberine class of BIAs, is present in species of each Ranunculales family, while the benzophenanthridine class, including the antimicrobial sanguinarine, is specific to the Papaveraceae family, and biosynthetic genes emerged after the split with the Ranunculaceae family 110 MYA but before the split of Papaveraceae species at 77 MYA. The phthalideisoquinoline noscapine and morphinan class of BIAs are exclusive to the opium poppy lineage. The morphine biosynthesis evolved more recently than 18 MYA in the genus *Papaver*. The major differences of medicinal BIAs between Papaveraceae and related families partially explain the uniqueness of its ethnomedicinal uses, and the material basis of therapeutic efficacies of different Papaveraceae genera also varies greatly [28]. In Papaveraceae (38 genera, >700 species), three categories showed a clustered structure. Thirty-two and seventeen *Corydalis* ethnomedicinal species are used for circulatory and nervous system diseases, respectively [3]. Accordingly, *Corydalis* components showed vascular relaxation effect [47] and neuroprotective effect [48] *in vitro* and/or *ex vivo*. Analgesia is one of the most important effects of *Corydalis* components, which are relatively nonaddictive and of low tolerance as compared to other analgesics [49]. Fifty-three out of 126 Papaveraceae species are used by China ethnic minorities against hepatobiliary diseases (Table S2), and *Corydalis* and *Meconopsis* species are salient in this treatment [20]. For instance, in Tibetan medicine, the whole plant of *Corydalis adunca* is used against liver-gallbladder sthenic heat [3], the root tuber is used against cholelithiasis, and the aboveground part is effective against biliary anorexia. In Tibetan medicine, the whole plant and flower of *Meconopsis quintuplinervia* are used against hepatitis, liver heat, and cholecystitis; in Qiang medicine, its whole plant and flower are used against jaundice. It should be pointed out that the overdispersion therapeutic categories are not trivial, as they may show a clustered structure at the subfamily, tribe, and/or genus level, or possibly because the relevant therapeutic compounds are ubiquitous throughout the whole family.

## 5. Conclusion

In conclusion, we compiled traditional ethnomedicinal uses of 551 Chinese species belonging to five families of Ranunculales from the Dictionary of Chinese Ethnic Medicine and recent literature, and the species-level phylogeny of angiosperms was used to analyze the phylogenetic signals of ethnomedicinal plants by calculating NRI and NTI. At the order level, the NRI results disclosed a clustered structure for eight therapeutic categories, that is, poisoning/intoxication, circulatory, gastrointestinal, eyesight, oral, pediatric, skin and urinary disorders, and the overdispersion for other seven categories; the NTI metric identified the clustered structure for all 15 categories. The NRI and NTI values were statistically significant in five and 11 categories, respectively. *Mahonia eurybracteata* of Berberidaceae has therapeutic effects on all categories. The most studied phytometabolites of Ranunculales include BIA, flavonoid, terpenoid, saponin, and lignan. [28]. The compound basis corresponding to the traditional efficacy is the focus of future exploration. By figuring out the phylogenetic distribution of therapeutic effects of Ranunculales ethnomedicinal plants, we illustrate the importance of quantitative phylogenetic methods in mining potential phytomedicine. The traditional medicinal knowledge could/should be scrutinized within the phylogenetic framework, making them not only important to local health and livelihoods, but also beneficial to the global health and sustainability. The protection of medicinal plant resources must be strengthened for better ecosystem service; protecting ecological environment is a basic national/global policy and is indispensable for human survival and welfare. To discover lead compounds with versatile bioactivities, in the near future, the ethnobotanical resources can be evaluated expansively by correlating large-scale phylogeny, spatial/geographic data, phytochemistry, and ethnopharmacology cues.

## Figures and Tables

**Table 1:** Phylogenetic clustering of Chinese ethnomedicinal plants of Ranunculales used for 15 diseases.

Disease category	NRI	*p* value	NTI	*p* value	No. of species
Poisoning/intoxication	0.2827673	0.382	2.5808124	0.001^*∗*^	105
Circulatory disease	0.3276161	0.357	0.9576053	0.176	80
Gastrointestinal disease	0.8993280	0.178	2.3439243	0.012^*∗*^	**343**
Nervous system disease	−1.2169750	0.900	1.6407281	0.046^*∗*^	78
Eye disease	5.3230108	0.001^*∗*^	3.1022603	0.001^*∗*^	110
Other/general disease	−0.4234927	0.644	2.9096083	0.005^*∗*^	**435**
Hepatobiliary disease	−2.0673037	0.987	1.1511975	0.134	183
Musculoskeletal disease	−1.8124459	0.978	2.0655373	0.024^*∗*^	**389**
Oral disease	2.9318791	0.006^*∗*^	2.3750417	0.009^*∗*^	132
Ear/nose/throat disease	−1.3491137	0.922	2.5423622	0.007^*∗*^	181
Pediatric disease	3.0196803	0.006^*∗*^	1.2504461	0.108	73
Reproductive system disease	−1.9141517	0.985	2.3016542	0.011^*∗*^	144
Respiratory disease	−3.7510929	1.000	0.4516397	0.340	239
Skin disease	2.0247475	0.025^*∗*^	2.1795913	0.023^*∗*^	**348**
Urinary disease	1.8995941	0.048^*∗*^	2.3121644	0.012^*∗*^	124

Note: NRI, net relatedness index; NTI, nearest taxon index; ^*∗*^*p* < 0.05, statistically significant; four categories with the largest number of species are shown in bold.

**Table 2:** Chinese ethnomedicinal species with no less than 10 categories of therapeutic efficacy.

Species	No. of therapeutic categories
Ranunculaceae: *Aconitum carmichaelili*	13
*Aconitum flavum*	10
*Aconitum kusnezoffii*	10
*Aconitum naviculare*	11
*Aconitum scaposum*	13
*Anemone hupehensis*	10
*Anemone rivularis*	12
*Anemone rivularis var. flore-minore*	10
*Anemone vitifolia*	11
*Clematis armandii*	12
*Clematis chinensis*	12
*Clematis hexapetala*	12
*Clematis manshurica*	12
*Coptis chinensis*	12
*Coptis chinensis var. brevisepala*	12
*Coptis deltoidea*	12
*Coptis omeiensis*	12
*Coptis quinquesecta*	12
*Coptis teeta*	12
*Nigella glandulifera*	10
*Nigella glandulifera*	10
*Ranunculus japonicus*	10
*Semiaquilegia adoxoides*	12
*Thalictrum acutifolium*	10
*Thalictrum alpinum var. elatum*	12
*Thalictrum cultratum*	11
*Thalictrum delavayi*	11
*Thalictrum foliolosum*	11
*Thalictrum ramosum*	11
*Thalictrum reticulatum*	11
*Thalictrum trichopus*	11
Berberidaceae: *Berberis amurensis*	12
*Berberis dasystachya*	10
*Berberis diaphana*	12
*Berberis heteropoda*	10
*Berberis jamesiana*	10
*Berberis julianae*	11
*Berberis poiretii*	13
*Berberis pruinose*	10
*Berberis vernae*	13
*Berberis vulgaris*	11
*Berberis wilsoniae*	12
*Epimedium acuminatum*	10
*Epimedium sagittatum*	10
*Mahonia bealei*	14
*Mahonia eurybracteata*	15
*Mahonia fortune*	14
*Mahonia gracilipes*	14
Menispermaceae: *Arcangelisia gusanlung*	11
*Cyclea hypoglauca*	10
*Stephania cepharantha*	11
*Stephania hernandiifolia*	10
*Stephania kuinanensis*	10
*Stephania kwangsiensis*	11
*Stephania kwangsiensis*	11
*Stephania micrantha*	10
*Stephania tetrandra*	10
*Tinospora capillipes*	13
*Tinospora crispa*	10
*Tinospora sagittate*	13
*Tinospora sinensis*	13
Lardizabalaceae: *Akebia trifoliata*	10
*Akebia trifoliata var. australis*	11
*Sargentodoxa cuneata*	10
Papaveraceae: *Corydalis edulis*	10
*Eomecon chionantha*	10
*Macleaya cordata*	10
*Macleaya microcarpa*	10

## Data Availability

The data generated or analyzed during this study are included within the article.

## Abbreviations

BBI: Bisbenzylisoquinoline
BIA: Benzylisoquinoline alkaloid
MNTD: Mean nearest taxon distance
MPD: Mean pairwise phylogenetic distance
NRI: Net relatedness index
NTI: Nearest taxon index
TCM: Traditional Chinese medicine.

## Supplementary Materials

Supplementary Materials Table S1: detailed efficacy matrix of Ranunculales ethnomedicinal plants. Table S2: phylogenetic clustering of Chinese ethnomedicinal plants of five major Ranunculales families used for 15 diseases. R language codes for the construction of the tree as well as calculation of the NRI and NTI.

## References

[1] Li L., Zhang B., Xiao P. (2016). Patterns and environmental determinants of medicinal plant: vascular plant ratios in Xinjiang, Northwest China. *PLoS One*.

[2] Defossez E., Pitteloud C., Descombes P. (2021). Spatial and evolutionary predictability of phytochemical diversity. *Proceedings of the National Academy of Sciences*.

[3] Jia M. R., Zhang Y. (2016). *Dictionary of Chinese Ethnic Medicine*.

[4] Wang W. H., Guan X. L. (2015). *Botany*.

[5] Zaman W., Ye J., Saqib S. (2021). Predicting potential medicinal plants with phylogenetic topology: inspiration from the research of traditional Chinese medicine. *Journal of Ethnopharmacology*.

[6] Zaman W., Ye J. F., Ahmad M., Saqib S., Shinwari Z. K., Chen Z. (2022). Phylogenetic exploration of traditional Chinese medicinal plants: a case study on Lamiaceae (angiosperms). *Pakistan Journal of Botany*.

[7] Zaman W., Ahmad M., Zafar M. (2020). The quest for some novel antifertility herbals used as male contraceptives in district Shangla, Pakistan. *Acta Ecologica Sinica*.

[8] Saslis-Lagoudakis C. H., Savolainen V., Williamson E. M. (2012). Phylogenies reveal predictive power of traditional medicine in bioprospecting. *Proceedings of the National Academy of Sciences*.

[9] Yessoufou K., Daru B. H., Muasya A. M. (2015). Phylogenetic exploration of commonly used medicinal plants in South Africa. *Molecular Ecology Resources*.

[10] Souza E. N. F., Williamson E. M., Hawkins J. A. (2018). Which plants used in ethnomedicine are characterized? Phylogenetic patterns in traditional use related to research effort. *Frontiers of Plant Science*.

[11] Wati R. K., de Graaf E. F., Bogarín D. (2020). Antimicrobial activity of necklace orchids is phylogenetically clustered and can be predicted with a biological response method. *Frontiers in Pharmacology*.

[12] Fabricant D. S., Farnsworth N. R. (2001). The value of plants used in traditional medicine for drug discovery. *Environmental Health Perspectives*.

[13] Jan H. A., Jan S., Bussmann R. W., Wali S., Sisto F., Ahmad L. (2020). Complementary and alternative medicine research, prospects and limitations in Pakistan: a literature review. *Acta Ecologica Sinica*.

[14] Li Y., Kong D., Fu Y., Sussman M. R., Wu H. (2020). The effect of developmental and environmental factors on secondary metabolites in medicinal plants. *Plant Physiology and Biochemistry*.

[15] Zaman W., Saqib S., Ullah F., Ayaz A., Ye J. (2020). COVID‐19: Phylogenetic approaches may help in finding resources for natural cure. *Phytotherapy Research*.

[16] Chen Z.-D., Yang T., Lin L. (2016). Tree of life for the genera of Chinese vascular plants. *Journal of Systematics and Evolution*.

[17] Lu L.-M., Mao L.-F., Yang T. (2018). Evolutionary history of the angiosperm flora of China. *Nature*.

[18] Hu H. H., Liu B., Liang Y. S. (2020). An updated Chinese vascular plant tree of life: phylogenetic diversity hotspots revisited. *Journal of Systematics and Evolution*.

[19] Hao D. C., Yang L. (2016). Drug metabolism and disposition diversity of *Ranunculales phytometabolites*: a systems perspective. *Expert Opinion on Drug Metabolism & Toxicology*.

[20] Hao D.-C., Xiao P.-G., Liu C. (2018). Traditional Tibetan medicinal plants: a highlighted resource for novel therapeutic compounds. *Future Medicinal Chemistry*.

[21] Ren K., Zhang W., Wu G. (2016). Synergistic anti-cancer effects of galangin and berberine through apoptosis induction and proliferation inhibition in oesophageal carcinoma cells. *Biomedicine & Pharmacotherapy*.

[22] Li P., Liu S., Liu Q. (2019). Screening of acetylcholinesterase inhibitors and characterizing of phytochemical constituents from Dichocarpum auriculatum (Franch.) WT Wang and P. K. Hsiao through UPLC-MS combined with an acetylcholinesterase inhibition assay *in vitro*. *Journal of Ethnopharmacology*.

[23] Hao D.-C., Li P., Xiao P.-G., He C.-N. (2021). Dissection of full-length transcriptome and metabolome of Dichocarpum (Ranunculaceae): implications in evolution of specialized metabolism of Ranunculales medicinal plants. *PeerJ*.

[24] Kembel S. W., Cowan P. D., Helmus M. R. (2010). Picante: R tools for integrating phylogenies and ecology. *Bioinformatics*.

[25] Webb C. O., Ackerly D. D., McPeek M. A., Donoghue M. J. (2002). Phylogenies and community ecology. *Annual Review of Ecology and Systematics*.

[26] Lu A. M., Tang Y. C. (2020). *The Origin and Evolution of Primitive Angiosperms*.

[27] Chen Z. D., Lu A. M., Liu B., Ye J. F. (2020). *Tree of Life for Chinese Vascular Plants*.

[28] Hao D. C. (2018). *Ranunculales Medicinal Plants: Biodiversity, Chemodiversity and Pharmacotherapy*.

[29] Xie S.-N., Yuan Q., Yang Q.-E. (2017). *Dichocarpum lobatipetalum* and *D. malipoense* (Ranunculaceae) are both merged with *D. hypoglaucum*. *Phytotaxa*.

[30] Hao D.-C., Xiao P.-G., Ma H.-Y., Peng Y., He C.-N. (2015). Mining chemodiversity from biodiversity: pharmacophylogeny of medicinal plants of Ranunculaceae. *Chinese Journal of Natural Medicines*.

[31] Xiao P. G., Wang L. W., Lv S. J. (1986). Statistical analysis of the ethnopharmacologic data based on Chinese medicinal plants by electronic computer I. Magnoliidae. *Chinese Journal of Integrated Traditional and Western Medicine*.

[32] Kunwar R. M., Mahat L., Acharya R. P., Bussmann R. W. (2013). Medicinal plants, traditional medicine, markets and management in far-west Nepal. *Journal of Ethnobiology and Ethnomedicine*.

[33] Sobhani Z., Akaberi M., Amiri M. S., Ramezani M., Emami S. A., Sahebkar A. (2021). Medicinal species of the genus Berberis: a review of their traditional and ethnomedicinal uses, phytochemistry and pharmacology. *Pharmacological Properties of Plant-Derived Natural Products and Implications for Human Health*.

[34] Srivastava S., Srivastava M., Misra A., Pandey G., Rawat A. (2015). A review on biological and chemical diversity in Berberis (Berberidaceae). *EXCLI Journal*.

[35] Jia X.-J., Li X., Wang F., Liu H.-Q., Zhang D.-J. (2017). Berbamine exerts anti-inflammatory effects via inhibition of NF-*κ*B and MAPK signaling pathways. *Cellular Physiology and Biochemistry*.

[36] Belwal T., Giri L., Bhatt I. D., Rawal R. S., Pande V. (2017). An improved method for extraction of nutraceutically important polyphenolics from Berberis jaeschkeana C.K. Schneid. fruits. *Food Chemistry*.

[37] Chignell C. F., Sik R. H., Watson M. A., Wielgus A. R. (2007). Photochemistry and photocytotoxicity of alkaloids from goldenseal (*Hydrastis canadensis* L.) 3: effect on human lens and retinal pigment epithelial cells. *Photochemistry and Photobiology*.

[38] Imenshahidi M., Hosseinzadeh H. (2019). Berberine and barberry (*Berberis vulgaris*): a clinical review. *Phytotherapy Research*.

[39] Malhotra B., Kulkarni G. T., Dhiman N. (2021). Recent advances on *Berberis aristata* emphasizing berberine alkaloid including phytochemistry, pharmacology and drug delivery system. *Journal of Herbal Medicine*.

[40] Jiang Y., Liu M., Liu H., Liu S. (2020). A critical review: traditional uses, phytochemistry, pharmacology and toxicology of Stephania tetrandra S. Moore (Fen Fang Ji). *Phytochemistry Reviews*.

[41] Desgrouas C., Taudon N., Bun S.-S. (2014). Ethnobotany, phytochemistry and pharmacology of Stephania rotunda Lour. *Journal of Ethnopharmacology*.

[42] Kumar P., Kamle M., Mahato D. K. (2020). Tinospora cordifolia (Giloy): phytochemistry, ethnopharmacology, clinical application and conservation strategies. *Current Pharmaceutical Biotechnology*.

[43] Vinh L. B., Jo S. J., Nguyen Viet P. (2021). The chemical constituents of ethanolic extract from Stauntonia hexaphylla leaves and their anti-inflammatory effects. *Natural Product Research*.

[44] Li J., Du K., Liu D., Meng D. (2020). New nor-oleanane triterpenoids from the fruits of *Stauntonia brachyanthera* with potential anti-inflammation activity. *Natural Product Research*.

[45] Lu C., Fan G., Wang D. (2020). Akebia Saponin D ameliorated kidney injury and exerted anti-inflammatory and anti-apoptotic effects in diabetic nephropathy by activation of NRF2/HO-1 and inhibition of NF-kB pathway. *International Immunopharmacology*.

[46] Li Y., Winzer T., He Z., Graham I. A. (2020). Over 100 million years of enzyme evolution underpinning the production of morphine in the Papaveraceae family of flowering plants. *Plant Communications*.

[47] Zhou Z. Y., Zhao W. R., Shi W. T. (2019). Endothelial-dependent and independent vascular relaxation effect of tetrahydropalmatine on rat aorta. *Frontiers in Pharmacology*.

[48] Kim Y. J., Lim H.-S., Kim Y., Lee J., Kim B.-Y., Jeong S.-J. (2017). Neuroprotective effect of *Corydalis ternata* extract and its phytochemical quantitative analysis. *Chemical and Pharmaceutical Bulletin*.

[49] Deng A.-P., Zhang Y., Zhou L. (2021). Systematic review of the alkaloid constituents in several important medicinal plants of the genus Corydalis. *Phytochemistry*.

