# Supplementary material for: Distribution of Therapeutic Efficacy of Ranunculales Plants Used by Ethnic Minorities on the Phylogenetic Tree of Chinese Species
Source: Evid Based Complement Alternat Med. 2022 Jan 12;2022:9027727. doi: 10.1155/2022/9027727 (PMC8769838; doi:10.1155/2022/9027727)
Supplement: Supplementary Materials — Table S1: detailed efficacy matrix of Ranunculales ethnomedicinal plants. Table S2: phylogenetic clustering of Chinese ethnomedicinal plants of five major Ranunculales families used for 15 diseases. R language codes for the construction of the tree as well as calculation of the NRI and NTI. [file 9027727.f1.zip › 9027727.f1/submit Supple text,R language codes for the construction of tree as well as calculating NRI and NTI.docx]

R language codes for the construction of tree as well as calculating NRI and NTI

library(picante)

library(ape)

sample <- read.csv("species.csv", header=T)

tree <- read.tree("full_tree_461.tre")

tip.all <- tree$tip.label

tip.not <- tip.all[!tip.all%in%(sample[,1])]

length(tip.not)

tr.new <- drop.tip(tree,tip.not)

write.tree(tr.new,"phylo_zhang_MGM.tree")

library(picante)

tree <- read.tree("C:/Users/22841/Desktop/full_tree_461.tre")

mydata <- read.table("C:/Users/22841/Desktop/sample.txt",header = T)

COMM1 <- as.matrix(mydata,rownames.force=NA)

COMM <- match.phylo.comm(tree,COMM1)

phydist <- cophenetic(COMM$phy)

ses.mpd.result <- ses.mpd(COMM$comm,phydist,null.model="phylogeny.pool",abundance.weighted=F,runs=999)

ses.mpd.result

NRI <- -ses.mpd.result$mpd.obs.z

NRI

ses.mntd.result <- ses.mntd(COMM$comm, phydist, null.model = "phylogeny.pool",abundance.weighted = F, runs = 999)

ses.mntd.result

NTI <- -ses.mntd.result$mntd.obs.z

NTI
